# A novel ligand of calcitonin receptor reveals a potential new sensor that modulates programmed cell death

**DOI:** 10.1038/cddiscovery.2016.62

**Published:** 2016-10-10

**Authors:** SGB Furness, DL Hare, A Kourakis, AM Turnley, PJ Wookey

**Affiliations:** 1 Drug Discovery Biology Laboratory, Monash Institute of Pharmaceutical Science, 381 Royal Parade, Parkville, Victoria 3052, Australia; 2 Department of Pharmacology, Monash University, Wellington Road, Clayton, Parkville, Victoria 3800, Australia; 3 Department of Medicine, University of Melbourne, Austin Health, Level 7, Lance Townsend Building, Studley Road, Heidelberg, Victoria 3084, Australia; 4 Department of Anatomy and Neuroscience, Melbourne Brain Centre, University of Melbourne, Royal Parade, Parkville, Victoria 3010, Australia

## Abstract

We have discovered that the accumulation of an anti-calcitonin receptor (anti-CTR) antibody conjugated to a fluorophore (mAb2C4:AF568) provides a robust signal for cells undergoing apoptotic programmed cell death (PCD). PCD is an absolute requirement for normal development of metazoan organisms. PCD is a hallmark of common diseases such as cardiovascular disease and tissue rejection in graft *versus* host pathologies, and chemotherapeutics work by increasing PCD. This robust signal or high fluorescent events were verified by confocal microscopy and flow cytometry in several cell lines and a primary culture in which PCD had been induced. In Jurkat cells, GBM-L2 and MG63 cells, the percentage undergoing PCD that were positive for both mAb2C4:AF568 and annexin V ranged between 70 and >90%. In MG63 cells induced for the preapoptotic cell stress response (PACSR), the normal expression of *α*-tubulin, a key structural component of the cytoskeleton, and accumulation of mAb2C4:AF568 were mutually exclusive. Our data support a model in which CTR is upregulated during PACSR and recycles to the plasma membrane with apoptosis. In cells committed to apoptosis (*α*-tubulin negative), there is accumulation of the CTR-ligand mAb2C4:AF568 generating a high fluorescent event. The reagent mAb2C4:AF568 effectively identifies a novel event linked to apoptosis.

## Introduction

Programmed cell death (PCD) is an absolute requirement for development in the foetus with dysregulation of PCD during embryogenesis, leading to a range of developmental defects, while in the adult, dysregulation is a key step in ontogeny of cancer.

Several distinct types of PCD have been defined, including apoptosis, necroptosis and autophagy, as well as several other forms.^1–3^ Necroptosis is currently viewed as the dominant mode in forms of inflammation.^4^ The intracellular pathways that characterize different forms of PCD and their cross interactions have been reviewed recently.^5,6^ Early events in PCD include externalization of phosphatidylserine to which annexin V binds and signals to phagocytes.^7^ However, imaging with annexin V is also useful to visualize inflammation and cell stress in the context of cardiovascular disease.^8^

A variety of both PCD promoting and PCD inhibiting cytokines have been well characterized that act through the receptor tyrosine kinase family of cell surface receptors. On the other hand, G protein-coupled receptors (GPCRs) are the largest family of cell surface receptors and are present in all metazoan organisms. Signalling from a range of GPCRs has been shown to modulate the cellular commitment to apoptosis. Here we present data consistent with a role for the calcitonin receptor (CTR) in events associated with PCD.

CTR is the least divergent member of the secretin-like family of GPCRs^9^ with a high proportion of conserved amino acids in vertebrates (e.g., >55% between aves and amphibia).

In mammals, CTR is widely expressed during foetal^10,11^ and perinatal^12,13^ development, in adult^14,15^ by lymphocytes,^16–18^ in inflammation associated with wound healing^15^ and cardiovascular disease^19,20^ and in several cancers.^15,21–25^ CTR is the only characterized receptor for calcitonin (CT) and also serves as the signalling protomer in the heteromeric amylin receptor (CTR/RAMP1). CTR is best characterized as coupling to the stimulatory G*α* subunit to increase adenylate cyclase activity and activate downstream cAMP sensors PKA and Epac but has also been shown to couple to intracellular calcium mobilization and extracellular-regulated kinase (ERK) phosphorylation. Activation of the CTR by both endogenous and exogenous agonists has been shown to modulate apoptosis in a number of settings. In osteoclasts cultured *in vitro*, exogenous teleost CT protects from chemically induced apoptosis^26,27^ through the inhibition of caspase activation^27^ via cAMP^26^ and pERK pathways.^27^ Generally, phosphorylation of ERK1/2 promotes cell survival; however, activation can also be proapoptotic.^28^ In prostate cancer cell lines, exogenous CT also protects from chemically induced apoptosis^29,30^ again via inhibition of caspase activation.^30^ In these cells, knockdown of CTR induces apoptosis,^31^ suggesting autocrine activation of CTR. In contrast, apoptosis of p53-deficient lymphomas can be induced by amylin acting via the CTR.^32,33^ Consistent with this finding, induction of apoptosis by CT has also been observed in HEK 293 cells transfected with CTR.^34^ CTR is also expressed in quiescent muscle satellite cells where it acts to protect these cells from apoptosis,^35^ although the ligand is unknown.

We have recently described the increased expression of CTR in the brain tumour glioblastoma.^25^ In an effort to understand the role of CTR in this type of cancer, we developed a novel CTR ligand consisting of a fluorescently coupled anti-CTR monoclonal antibody directed against an extracellular, N-terminal epitope (mAb2C4:AF568). Although this reagent was easily able to detect CTR on recombinant cell lines (see Supplementary Figures S1 and S3 for validation of this reagent) and was highly specific for CTR, we found that CTR expression in the GBM cell line A-172 to be below the level that could be detected. This was despite the fact that ligands of CTR stimulated these cells.^25^ During these studies, we observed a number of small, rounded cells with condensed nuclei and appeared to be apoptotic that were brightly fluorescent with accumulation of mAb2C4:AF568, prompting us to investigate the potential role of CTR in PCD.

## Results

In a previous publication, experiments for the validation of two anti-CTR antibodies (mAb9B4: extracellular epitope 4; mAb1H10: intracellular epitope 1) were presented.^25^ MAb2C4 (IgG1) also binds a peptide equivalent to epitope 4, and data supporting its specificity in immunoblots and flow cytometry are shown in Supplementary Figure S1.

**Figure 1:**
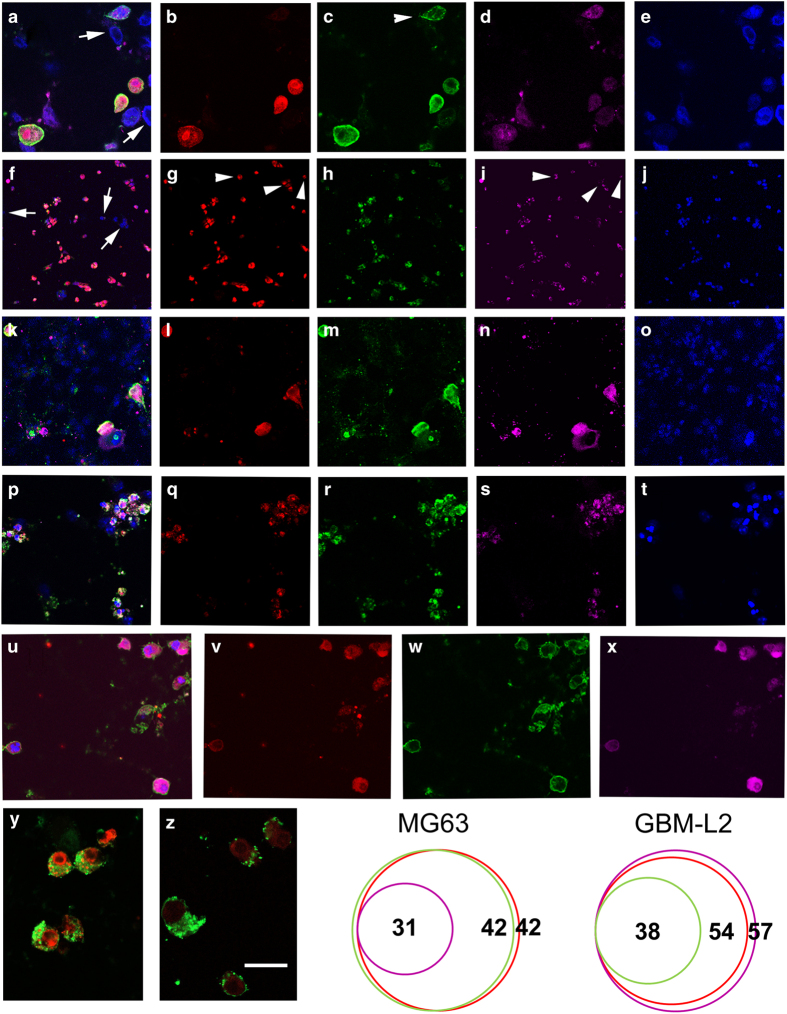
Cell lines induced to undergo apoptosis with treatment of staurosporine for 19 h, representative images of >20 separate experiments. Treated cell cultures were live stained with mAb2C4:AF568 and annexin V:AF488 prior to fixation and staining with anti-cleaved caspase 3 antibody. (**a**–**e**) A172 cells treated with 1 *μ*M staurosporine: (**a**) merged image, arrows indicate examples of nuclei from unaffected cells; (**b**) mAb2C4:AF568; (**c**) annexin V:AF488, the arrowhead indicates an example with no uptake of mAb2C4:AF568; (**d**) caspase 3; and (**e**) DAPI (4,6-diamidino-2-phenylindole). (**f**–**j**) GBM-L2 cells treated with 1 *μ*M staurosporine: (**f**) merged image, arrows indicate examples of nuclei from unaffected cells; (**g**) mAb2C4:AF568; (**h**) annexin V:AF488; (**i**) caspase 3; and (**j**) DAPI. The arrowheads in panels (**g** and **i**) indicate apoptotic cells for which there is little or no annexin V:AF488 signal. (**k**–**o**) COS-7 cells treated with 1 *μ*M staurosporine: (**k**) merged image; (**l**) mAb2C4:AF568; (**m**) annexin V:AF488; (**n**) caspase 3; and (**o**) DAPI. (**p**–**t**) Primary mouse neural precursor cells treated with 0.1 *μ*M staurosporine: (**p**) merged image; (**q**) mAb2C4:AF568; (**r**) annexin V:AF488; (**s**) caspase 3; and (**t**) DAPI. (**u**–**x**) MG63 cells were treated with 1 *μ*M staurosporine: (**u**), a merged image; (**v**) mAb2C4:AF568; (**w**) annexin V:AF488; and (**x**) cleaved caspase 3. MG63 cells were treated with 1 *μ*M staurosporine showing merged images with annexin V:AF488 plus ((**y**), HFEs) and minus ((**z**), autofluorescence) mAb2C4:AF568. Venn diagrams of relative cell counts for the cell line MG63 and GBM-L2, and overlap for annexin V, mAb2C4:AF568 and caspase 3. The calibration bar shown in panel (**z**) represents (**a**–**e**) 25 *μ*m; (**f**–**j**) 80 *μ*m; (**k**–**o**) 25 *μ*m; (**p**–**t**) 60 *μ*m; (**u**–**x**) 25 *μ*m; (**y** and **z**) 20 *μ*m.

To test whether a change in receptor cycling did indeed accompany PCD (example of binding and uptake shown for 0 and 60 min in Supplementary Figure S2) in a number of different cell types, we first treated the A-172 cell lines with staurosporine to induce PCD (an inhibitor of protein kinases^36^). Although staurosporine inhibits protein kinases, induction of PCD occurs through caspase-dependent and -independent pathways.^37^ These cells were then subjected to live staining (Supplementary Information) for 30 min with our novel CTR ligand, mAb2C4:AF568, prior to fixation. Representative images are shown in Figures 1a–e with live co-staining with the conventional marker annexin V and staining postfixation to detect the executioner enzyme, cleaved caspase 3. The A-172 cell line is not generally regarded as a good model for GBM (cultured with serum and non-tumorigenic), and as we had originally made the observation that CTR is upregulated in primary tumours including glioma stem cells,^25^ we sought to confirm our results in the primary high-grade glioma line GBM-L2 (Figures 1f–j) that are representative of glioma stem cells^38,39^ and are grown serum-free. We also chose two representative cell lines that do not normally express CTR on the cell surface and indeed do not show pharmacological responses: Cos-7 kidney cells and, later for experiments with flow cytometry, the Jurkat T-cell lymphoma cell line. We examined the Cos-7 cell line induced for PCD with staurosporine, and the results are shown in Figures 1k–o. Next we tested a primary culture of mouse neuroblasts^40^ treated with staurosporine (Figures 1p–t) and demonstrated that apoptotic cells also bound annexin V, accumulated MAb2C4:AF568 and expressed activated caspase 3. MG63 cells (Figures 1u–x) also responded similarly. Following live staining with mAb2C4:AF568 and annexin V:AF488 (merged image, Figure 1u), high fluorescence events (HFEs) associated with internalization of mAb2C4:AF568 (Figure 1v) correlated strongly with binding of annexin V (green, Figure 1w). Activated caspase 3 is shown in Figure 1x. Together with a shrunken cytoplasm and condensed nuclei, these positive markers demonstrate that these cells are apoptotic.

Two further panels (y and z) have been included to demonstrate that the HFE (uptake of mAb2C4:AF568) is much more intense than autofluorescence associated with dying cells.

We quantified the data (by counting co-stained cells) of MG63 and GBM-L2 cells and present the data as Venn diagrams. For the MG63 cell line (Figure 1), we observed almost complete congruence between mAb2C4:AF568 and annexin V:AF488 staining, but only 74% also expressed detectable caspase 3. In the GBM-L2 cell line, we observed almost complete congruence between MAb2C4:AF568 and caspase 3 staining (Figure 1) but only 70% bound detectable levels of annexin V.

**Figure 2:**
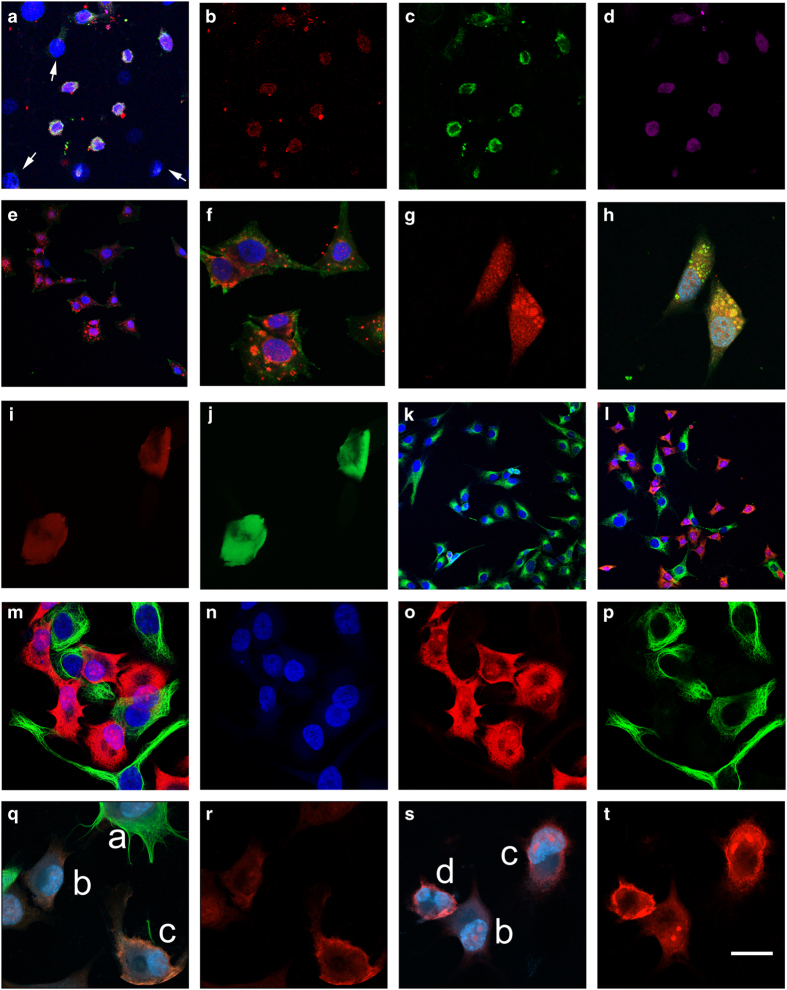
MG63 cells were treated with cytotoxins for 19 h to induce PCD. Live staining with mAb2C4:AF568 and annexin V:AF488, was followed by fixation and staining with additional primary antibodies (caspase 8, LC3B or *α*-tubulin) followed by specific species or isotype secondary antibody:AlexaFluors. (**a**–**d**) MG63 cells were treated with 1 *μ*M staurosporine: (**a**), a merged image, with arrows indicating examples of nuclei from unaffected cells; (**b**) mAb2C4:AF568; (**c**) annexin V:AF488; and (**d**) cleaved caspase 8. (**e** and **f**) merged images of MG63 cells were treated with 50 *μ*M etoposide+30 *μ*M necrostatin-1 (DAPI (4,6-diamidino-2-phenylindole), blue; mAb2C4:AF568, red; annexin V:AF488, green). (**g** and **h**) mAb2C4:AF568 (red channel) and merged image of MG63 cells treated with 50 *μ*M chloroquine to induce autophagy (DAPI, blue; mAb2C4:AF568, red; LC3B, green). (**i** and **j**) MG63 cells treated with tumor necrosis factor *α* and zVAD-fms underwent necroptosis (mAb2C4:AF568, red; annexin V, green). (**k**) MG63 cells were untreated as controls (DAPI, blue; *α*-tubulin, green). (**l**–**p**) MG63 cells were treated with 1% DMSO+1 *μ*M paclitaxel: (**l**, DAPI, blue; *α*-tubulin, green; mAb2C4:AF568, red) induced into the PACSR. (**q**–**t**) separate experiment with cells treated with 1% DMSO+1 *μ*M paclitaxel: (DAPI, blue; mAb2C4:AF568, red; *α*-tubulin, pseudo-green) The calibration bar shown in (**t**) represents (**a**–**e**, **k** and **l**) 25 *μ*m and (**f**–**j**, **m**-**t**) 10 *μ*m.

For more extensive studies (>20 independent experiments) and in order to understand some of the biology associated with this response and accumulation of mAb2C4:AF568, we chose to study in more detail the osteosarcoma cell line MG-63, which is similar to the A-172 cell line that expresses low but functional levels of CTR.^41^ Following induction of apoptosis for 19 h with 1 *μ*M staurosporine and live staining with the novel reagent mAb2C4:AF568, we observed accumulation of this CTR ligand specifically in cells positive for the expression of activated caspase 8 (Figure 2d) but not for activated caspase 9 (Supplementary Figure S4q), indicating extrinsic apoptosis by death receptors.^1^ In dying cells, localization of mAb2C4:AF568 in the cytoplasm was often concentrated in the perinuclear region (enlarged image, Supplementary Figure S2a) in contrast to annexin V binding to the plasma membrane. In similar experiments but with live staining of an isotype control (IgG1, mAb9E10:AF647, Supplementary Figure S1d), there was little evidence of internalization of this probe (shown in Supplementary Figure S4c). Further controls are displayed in Supplementary Figure S4. There appeared to be some overlap in the cytoplasmic localization of mAb2C4:AF568 and LAMP-1, a lysosomal marker (Supplementary Figure 4h).

MG63 cells shown at low and high magnification (Figures 2e and f) had been treated with 50 *μ*M etoposide plus 30 *μ*M necrostatin-1, which resulted in a flattened regular morphology with cytoplasmic inclusions loaded with mAb2C4:AF568 and are consistent with large vacuoles characteristic of autophagy.

In Figures 2g and h are shown images of MG63 cells following treatment with 50 *μ*M chloroquine, which induces autophagy. In many instances, mAb2C4:AF568 (red) co-localises with LC3B-positive (green) autophagosomes yielding a high fluorescent signal.

Necroptosis can be induced with TNF*α* and the caspase inhibitor zVAD-fms, and we found several instances of swollen necrotic cells in which there was relatively weak fluorescence associated with mAb2C4:AF568 (red) and annexin V:AF488 (green) (Figures 2i and j).

Dimethyl sulphoxide (DMSO) has been proposed to extend the preapoptotic cell stress response (PACSR) in hepatocytes^42,43^ or, alternatively, to promote differentiation with growth arrest at G0/G1 in pre-T human lymphoid cells.^44^ Untreated MG63 cells, as shown in Figure 2k, express *α*-tubulin (green, mAb2C4:AF568-negative) and divide normally. To induce the PACSR, MG63 cells were treated with 1% DMSO (Supplementary Figures S4r–u) or 1% DMSO plus 1 *μ*M paclitaxel (Figures 2l–t). Paclitaxel stabilizes the microtubules, inhibits cell cycle progression (G2-M transition) and promotes apoptosis. In Figure 2l is an image captured at low magnification showing distinct subpopulation of cells that stain exclusively for *α*-tubulin filaments (green) or uptake of mAb2C4:AF568 (red), and the latter display reduced cytoplasm and smaller nuclei, as expected for apoptotic cells. These exclusive cell populations are better viewed at high magnification (Figures 2m–q). In these panels, mAb2C4:AF568 (red), *α*-tubulin (green) and DAPI (blue) and the merged images are shown. Using a Zeiss LSM800 confocal microscope with airyscan, high resolution images are shown in Figures 2q–t of dying cells with strong binding of mAb2C4:AF568 to the outside domain of the cell (Figures 2r and t), supporting the interpretation that CTR is externalized during the PCD process.

**Figure 3:**
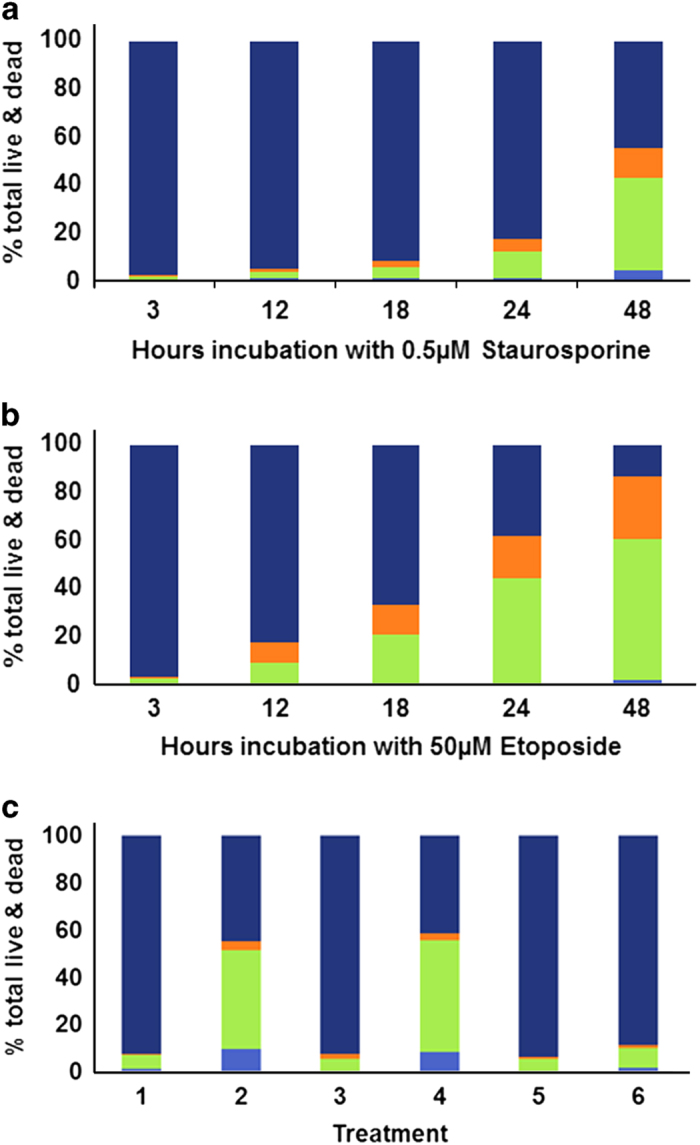
Flow cytometry (FACS analysis) of Jurkat cells treated with (**a**) 0.5 *μ*M staurosporine or (**b**) 50 *μ*M etoposide. In (**c**), treatments for 24 h were (1) 10 *μ*M rapamycin; (2) 50 *μ*M etoposide; (3) 50 *μ*M etoposide plus pan caspase inhibitor z-VAD-fmk; (4) 15 ng/ml TRAIL; (5) 15 ng/ml TRAIL plus z-VAD-fmk; (6) untreated. (**a**–**c**) ■, Annexin V-positive; ■, Annexin V-positive and mAb2C4:AF568-positive; ■, mAb2C4:AF568-positive and ■; double-negative, viable cells.

Consistent with these data was the response of Jurkat cells when stained and analysed by flow cytometry (Figure 3). The advantage with flow cytometry is the capture of all cells undergoing PCD and analysis by fluorescence activated cell sorting (FACS). Several cytotoxins were chosen to distinguish between cytotoxin-specific effects and those arising from a commitment to PCD. Staurosporine and etoposide were initially chosen as the latter is an inhibitor of topoisomerase II (quite different from staurosporine), an action that leads to DNA damage and apoptosis.^45^ TRAIL^46^ activates the death receptor and also promotes PCD via a caspase-dependent pathway. Rapamycin activates mTOR^47^ and promotes autophagy in some cell lines.

We tested the time-dependent effects of staurosporine or etoposide. A small percentage of Jurkat cells treated with either staurosporine or etoposide bound annexin V alone, but 70–80% co-stained with mAb2C4:AF568 (Figures 3a and b). There was also an increasing population of singly stained (mAb2C4:AF568) cells with time, suggesting necroptosis or secondary necrosis. We also tested 24 h exposure (rapamycin, etoposide, TRAIL) and inhibiton of caspases with the pan-caspase inhibitor z-VAD-fmk (Figure 3c). These data demonstrate that the majority of PCD is apoptotic with a small side population of putative necrotic cells.

The isotype control used for FACS experiments was mAb9E10(IgG1):AF647 and experiments that demonstrate its binding to cMyc are shown in Supplementary Figure S1e.

As part of the normal receptor cycle, CTR is embedded in the endosomal membrane with the carboxyl terminus facing outwards into the cytoplasm. CTR endosomes participate in traffic within the cytoskeleton^48^ and are driven along the microtubules by molecular motors. Consistent with this model, we have identified both a PDZ docking motif (Supplementary Figure S1f) and arginine sequences (Supplementary Figure S1g) in the carboxyl domain of CTR, in which the arginines share similar spacing to arginines of the carboxyl domain of *α*2B-adrenergic receptor (*α*2B-AR).^49^ In the latter receptor, three of these arginines in the carboxyl domain (Arg437, Arg441 and Arg446) have been demonstrated to be important for the interaction with *α*-tubulin.^49^ CTR from all animal species identified so far share a PDZ docking motif, but the spacing of the arginines varies and they are absent in one species (*Jaculus jaculus*) identified so far. The interaction with *α*-tubulin via PDZ domains has been well established. We have presented further data with immunoblots (Supplementary Figure S1a, lanes 8–10) in support of the interaction of CTR with *α*-tubulin.

CTR, as detected by mAb2C4 on immunoblots is highly concentrated in the cytosolic fraction compared with the nuclear proteins or membrane proteins in both COS-7 and MG63 cell lines. The apparent MW is approximately 55 kD on PAGE-SDS gels (Supplementary Figure S1b), which corresponds to the predicted full-length unglycosylated protein. Further studies on this fraction will be aimed at confirming these predictions and defining the suborganelle in this fraction with which CTR is associated.

Increased CTR mRNA levels over basal levels were observed with staurosporine- or etoposide-treated cell lines HCT116 and the mutant HCT116 bax^−/−^bak^−/−^ as measured by qPCR (Supplementary Figure S5). The latter are resistant to apoptosis and therefore elevated expression of CTR mRNA was not the result of apoptotic events but rather results from cytotoxic insult. The upregulation with cytotoxic insult is in agreement with a previous study in which CTR mRNA was elevated in primary cultures of human astrocytes treated with the proinflammatory cytokines TNF*α* or IL1*β*.^50^

**Figure 4:**
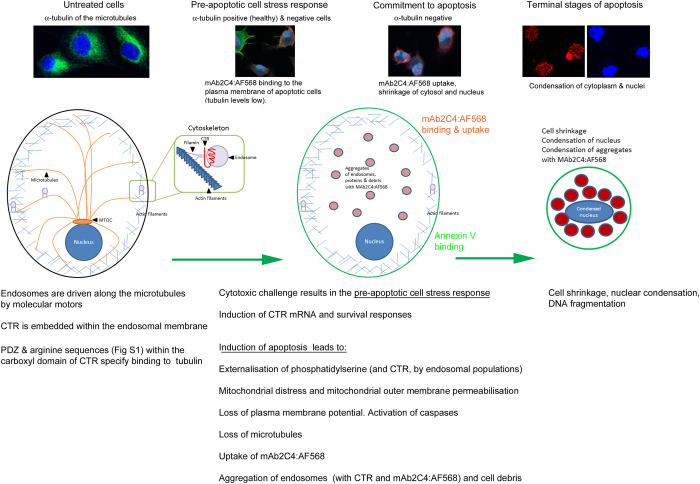
A model in which CTR is externalized during preapoptotic cell stress. In this model, mAb2C4:AF568 accumulates in the absence of *α*-tubulin within the lysosomes and with shrinkage of the cytoplasm results in a high fluorescent signal.

In Figure 4 is shown our model linking events following cytotoxic insult, which results in the PACSR, the upregulation of CTR (and CTR mRNA, Supplementary Figure S5), survival or capitulation to PCD and, in the case of the latter, externalization of phospatidylserine and CTR. These events are accompanied by activation of caspases and degradation of cellular components, including *α*-tubulin, with concomitant changes in morphology together with accumulation of mAb2C4:AF568.

## Discussion

In this study, we have demonstrated that an anti-hCTR antibody conjugate (mAb2C4:AF568) is accumulated following the period of PACSR, and as a result, an HFE is generated in shrinking apoptotic cells, which is useful for the detection of apoptosis. The HFE was clearly distinguished from autofluorescence often associated with dying cells.

This phenomenon was recorded in all cell lines tested so far and in a population of primary neuroblasts induced for PCD. This suggests a phenomenon in common with all cell types although this number is necessarily restricted in this study. This being the case, mAb2C4:AF568 (which we have called CalRexin) should be a useful reagent that faithfully monitors apoptosis and might prove useful for the selection of potential anticancer reagents using cancer cell lines some of which bind annexin V in the absence of PCD. Furthermore, we demonstrated co-localization of mAb2C4:AF568 fluorescence with LC3B-positive autophagosomes in MG cells induced with chloroquine. Further studies are aimed at the generalization of this observation.

CTR expression has been reported in all vertebrates investigated so far and in several examples of invertebrates although analysis and clear characterization of CTR is not exhaustive. It is possible that CTR is involved, as described in our model, in apoptosis throughout the animal kingdom and future studies should investigate examples within the invertebrates. This will probably require anti-epitope 4 CTR antibodies specific for each class of species that share high identity in epitope 4.

Our data demonstrate that the upregulation of CTR mRNA is in response to cytotoxic insult rather than the induction of PCD. Our data also clearly show that uptake of mAb2C4:AF568 is dependent on the induction of PCD rather than a response to a particular cytotoxin.

In data shown here, mAb2C4:AF568 accumulates into MG63 cells that no longer express *α*-tubulin, as an indicator of commitment to PCD. Caspase 6 is widely expressed in many organs^51^ and is regarded as one of the three (-3, -6 and -7) executioner caspases for apoptosis. One of its targets has been demonstrated to be *α*-tubulin^52^ where there is a target sequence close to the carboxyl end at which caspase 6 cleaves. Cleavage of this fragment leads to instability of microtubules.^52^ In our model, this executioner caspase (and perhaps others) is activated in MG63 cells undergoing the early phases of apoptosis, which results in the degradation of *α*-tubulin.

We propose that with the onset of apoptotic program CTR expression is upregulated and CTR inserted into intracellular membranes (e.g., endosomes) participates in the process of externalization of phosphatidylserine, which is thought to be mediated by a subpopulation of endosomes^53^ and results in externalization of CTR. We demonstrated the concentration of mAb2C4:AF568 in outer surface of several cells (Figure 2), which is consistent with the process of externalization during the apoptotic process. Such early apoptotic events might also determine the release of glycolytic enzymes^54^ and the generation of exosomes for the export cytoplasmic toxins.^55^

In the early stages of PCD, such an event would lead to the binding of mAb2C4:AF568 to CTR exposed on the plasma membrane followed by internalization to generate an HFE as described in this manuscript. Residual accumulation of mAb2C4:AF568 would occur in the absence of CTR recycling along microtubules (comprised of *α*- and *β*-tubulin) to the plasma membrane and concentration in autophagosomes and lysosomes (LAMP 1-positive). In shrinking apoptotic cells, mAb2C4:AF568 is often located in the perinuclear domain and staining overlaps staining with LAMP-1 antibody, which is used to identify lysosomes. The mutual exclusion of tubulin expression and internalization of mAb2C4:AF568 in mixed subpopulations of MG63 cells, the former apparently resistant through survival mechanisms and the latter undergoing commitment to apoptosis, supports this model.

Our discovery described here offers unique opportunities to explore the proposed role in apoptosis and autophagy. The definition of this role will shed light on whether CTR activity is proapoptotic or antiapoptotic or both, depending on the environmental conditions (see Introduction). Of interest here, in the context of cancer biology and p53-deficient tumours, CTR mediates the action of one of its ligands (amylin) on glycolysis and promotes apoptosis in thymic lymphoma cells.^32,33^ The outcomes of this study and our model suggest that agonists of CTR might also prove to represent a novel class of antitumour drugs that promote apoptosis in the context of the cancer microenvironment for particular classes of cancer cell genotypes.

Finally, the exposure of CTR on the cell surface of cells undergoing apoptosis and the binding of the ligand antibody conjugate might prove a useful strategy for imaging PCD *in vivo*. Such a utility, if proven successful, would have a major impact in medicine for imaging diseases and the efficacy of treatments *in vivo*.

## Materials and Methods

### Antibodies

These studies included the use of three mouse monoclonal anti-human CTR antibodies, two directed against an extracellular epitope (mAb46/08-2C4 (isotype IgG1, mAb46/08-2C4:AF568 is GenWay GWB-CALR01, San Diego, CA, USA) and mAb30/7-9B4^25^ (isotype IgG2A)) and the third against a cytoplasmic epitope (mAb31/01-1H10^19,20,24,25^ (isotype IgG2A)). Details of mAb9E10 (anti-cMyc, isotype IgG1) have been published elsewhere.^56^ Validation of mAb2C4 is presented in Supplementary Figure S1, and for comparison, similar data for mAb9B4, mAb1H10 and the anti-cMyc mAb9E10 are included. Further data for the validation of mAb9B4 and mAb1H10 have been published.^25^

For the confocal analysis and multilabelling immuno-fluorescence experiments, the antibodies used included the anti-CTR antibodies (above), the anti-cleaved caspase 3 antibody (no. 9579, Cell Signaling, Danvers, MA, USA) with goat anti-rabbit:AF635 (Thermo Fisher Scientific, Waltham, MA, USA) and the Annexin V Detection Kit (TACS Annexin V-biotin, Trevigen, Gaithersburg, MD, USA) with streptavidin:AF488 (Thermo Fisher Scientific). Other primary antibodies used with fixed cells included anti-cleaved caspases 8 (rabbit no. 9496, Cell Signaling) and 9 (rabbit no. 9505, Cell Signaling), LAMP-1 (rabbit no. 9091, Cell Signaling) and anti-*α*-tubulin (mouse IgG1, T6074, Sigma Aldrich, St Louis, MO, USA) antibodies. Secondary goat antibodies and AlexaFluor conjugates were purchased from Thermo Fisher Scientific.

The isotype control antibody mAb9E10 (anti-myc, IgG1^56^) was conjugated with the AF568 (for confocal experiments) using the same technique for mAb2C4 described below. The retention of binding activity of the mAb9E10 conjugate was demonstrated by FACS analysis (Supplementary Figure S1).

### Chemical conjugation of antibodies

Both anti-CTR monoclonal antibody mAb2C4 and anti-cMyc monoclonal mAb9E10 (isotype control) were conjugated to AlexaFluor succinylamide (NHS) esters (Thermo Fisher Scientific) using standard techniques. The method of conjugation is summarized as follows.

#### Buffer exchange for conjugation

Two millilitres of mAb2C4 (5.8 mg/ml in neutral citrate buffer) was concentrated to 0.5 ml using an Amicon Ultra 15 centrifugal filters (MWCO~10 kD, UFC901008, EMD-Millipore, Billerica, MA, USA). Zeba spin column (Thermo Fisher Scientific: 5 ml, sample volume 500*μ*l to 2 ml) were prepared for buffer exchange by washing column (1000×*g* for 2 min) with 100 mM HCO_3_^−^ (pH 8.3) four times. An aliquot of 0.5 ml of concentrated mAb2C4 (~11.6 mg in neutral citrate buffer) was loaded onto the Zeba column and centrifuged (1000×*g* for 2 min). The eluent was collected, and the concentration was determined on a Nanodrop1000 spectrophotometer (Thermo Fisher Scientific).

#### Chemical conjugation

The eluent was diluted to 1.9 ml with 100 mM HCO_3_^−^ (pH 8.3) and 1 mg in 100–200 *μ*l anhydrous DMF and AF568:NHS esters (Thermo Fisher Scientific A20103: lot number 1639203, 0.85 activity, MW=792) added dropwise with gentle vortexing. The molar ratio was 14 of NHS esters to 1 of mAb2C4. A 15 ml tube covered with alfoil was rotated slowly for 1 h at RT, after which 50 *μ*l of 1.5 M TEA (20% solution in 100 mM HCO_3_^−^ buffer) was added to halt the reaction. The solution was buffer-exchanged with Zeba spin columns preequilibrated with PBS (prefiltered using a 0.2 *μ*m filter). The 2.0 ml aliquot was loaded onto spin columns and the eluent was collected.

#### Purification of active conjugates

The 2 ml aliquot was chromatographed on a peptide-affinity column (peptide corresponding to epitope 4 of CTR and conjugated (linker maleimidocaproyl-*N*-hydroxysuccinimide) to thiopropyl sepharose 6B ~4 ml bed volume, prepared by Mimotopes (Clayton, VIC, Australia). Once loaded, the pump was stopped for 5 min to allow binding and then the affinity column was washed with PBS. The breakthrough contained unbound conjugate in which the binding determinants were most likely compromised. The bulk of the conjugated material bound to the column was eluted with 100 mM glycine buffer (pH 2.2) and 1 ml fractions were collected into 100*μ*l TRIS buffer (pH 9). Active fractions were concentrated with Amicon Ultra-15 centrifugal filter devices-10 K MWCO (15 ml: EMD-Millipore UFC901008), washed with PBS/1 mM EDTA (prefiltered 0.22 *μ*m) and concentrated to ~1 ml. Using the Nanodrop1000, the *λ*1 (560 nm) and *λ*3 (280 nm) were recorded and used to calculate the amount of protein, the percentage of recovery and the degree of labelling (DOL) with AF568:NHS esters for the antibody conjugate. The final DOL was 4.4 and the yield 36%. The protein profile was checked on an 8% acrylamide gels stained with Coomassie (not shown). This overall protocol as outlined for the conjugation of mAb2C4 produced consistent labelling of active mAb2C4:AF568.

### Cell culture

The human osteosarcoma cell line MG63 was cultured in MEM*α* (Thermo Fisher Scientific) plus 10% foetal bovine serum (FBS, Thermo Fisher Scientific). The human glioblastoma cell line A172^25^ and monkey kidney cell line Cos-7 were cultured in Dulbecco’s modified Eagle’s medium (Thermo Fisher Scientific) plus 10% FBS. The high-grade glioma cell line GBM-L2 was cultured in StemPro media (serum free, Thermo Fisher Scientific A10509-01). Cultured cells were incubated in a humidified 37 °C incubator with 5% CO_^2^_. MG63 cells, A172 cells and Cos-7 cells were cultured in four-well chamber slides (Nunc 154526, Lab Tek II) and GBM-L2 on CC-2 chamber slides (Nunc 154917, Thermo Fisher Scientific) and each chamber was seeded with 50 000 cells and cultured until 50–80% confluent. A range of concentrations of the cytotoxin staurosporine (final 10^−6^, 10^−7^ and 10^−8^ M, Sigma Aldrich) were incubated with cell lines for 19 h to induce apoptosis. Other cytotoxins used included 50 *μ*M etoposide (Sigma Aldrich), 10 *μ*M rapamycin (VETRANAL, Sigma Aldrich) and 15 ng/ml TRAIL (Abcam, Cambridge, UK) sometimes in combination with the pan-caspase inhibitor 5 *μ*g/ml z-VAD-fms (Santa Cruz, Dallas, TX, USA).

#### Preparation of the primary cultures of mouse neural precursor cells

Neural precursor cell (Neurosphere) cultures were established from the cortices of postnatal day 1 C57BL/6 mice (Biomedical Animal Facility, The University of Melbourne, Parkville, Victoria, Australia) and cultured in Neurobasal medium (Thermo Fisher Scientific) containing 2% B27 supplement (Thermo Fisher Scientific), 0.5 mM glutamine (Thermo Fisher Scientific), 20 ng/ml EGF (PeproTech, Rocky Hill, NJ, USA) and 10 ng/ml fibroblast growth factor-2 (FGF2) (PeproTech), essentially as previously described.^40^ For analysis, neurospheres were plated onto Matrigel-coated chamber slides, where they flattened and grew as adherent cells.

### Live staining for microscopy

Cell lines when grown on chamber slides were washed once in media and 2 *μ*g/ml of mAb2C4:AF568 or mAb9E10:AF568 was introduced with further incubation for 30 min. As a positive control, HFEs were detected following live stain with mAb2C4, fixation and detection with secondary anti-mouse antibody conjugates. Negative controls included mAb9E10:AF568 (IgG1) and unconjugated IgG1 controls (DAKO, Glostrup, Denmark, X0931; R&D Systems, Minneapolis, MN, USA, MAB002) in a live stain followed by fixation and detection with anti-mouse secondary antibodies conjugated to fluorophores (Thermo Fisher Scientific).

### Annexin binding

The chambers were washed in cold binding buffer and annexin V:biotin (Trevigen Inc.) was added for 15 min. After fixation, annexin V:biotin binding was detected using streptavidin:488 (Thermo Fisher Scientific).

### Immuno-staining for confocal microscopy

Chambers were then washed and fixed in 4% paraformaldehyde (ProSci Tech, Thuringowa, QLD, Australia)/PBS (pH 7.2) for 30 min. After washing once in PBS, then twice in PBS/1% Triton-X100 (Sigma Aldrich) and blocking (1% BSA (Roche, Basel, Switzerland), 5% NGS in PBS/1% Triton-X100) for 1 h at room temperature, the primary anti-caspases antibodies (5 *μ*g/ml) or mAb1H10 (4 g/ml) were incubated overnight at 6 °C in a humidified chamber. The following day, the chambers were washed three times in PBS, and the cells were incubated in the dark at room temperature for 60 min with the secondary antibody (goat anti-rabbit:AF635 (Thermo Fisher Scientific), 1 : 500 in PBS) and streptavidin:AF488 (Thermo Fisher Scientific). The slides were washed three times, and the chambers were removed prior to mounting with DAPI aqueous mount (Prolong Gold, Thermo Fisher Scientific) and dried at room temperature for several days in the dark.

### Confocal microscopy and multi-channel detection

The samples were imaged by confocal microscopy (Objectives ×20, x40, ×63) using a Zeiss Imager Z1/LSM 510 Meta confocal laser scanning system (Zeiss, Oberkocken, Germany) using the Zen software (Zeiss). Images (LSM format) were captured in a single focal plane (optical sections of 0.7 *μ*m nominal thickness), converted to TIFF files using LSM Image Browser (Zeiss) and the figures for the manuscript were compiled in Photoshop (Adobe version 6.0, San Jose, CA, USA). Some images (Figures 2G-J, Q-T) were captured using a Zeiss LSM800 with airyscan (CZI format) and converted to jpeg files with Image J (NIH, Bethesda, MD, USA).

### FACS analysis

Jurkat cells were cultured in RPMI/10%FBS/11 mM glucose in 75 cm^2^ or 175 cm^2^ flasks. The cells were subcultured and resuspended in RPMI/10% depleted FBS/11 mM glucose. On the same day, cytotoxins were added and incubated for various periods from 3 to 48 h. To harvest the cells, they were centrifuged for 5 min at 1300 r.p.m. in a Sorvall T6000 fitted with a H1000B head and washed in RPMI 1640.

While on ice, the cells were resuspended in required volume of FACS buffer (1× binding buffer (for annexin V)/0.1% BSA). Each well of a v-bottom FACS plate contained 1×10^7^cells/ml in 100 *μ*l of FACS buffer. The plate was spun for 3 min at 1300 r.p.m. to pellet the cells. Cells were then blocked (on ice for 20 min) in 100 *μ*l block (1× binding buffer/5%BSA) and resuspended gently, then centrifuged for 3 min at 1300 r.p.m. The supernatant was removed and live stained with 2 *μ*g/ml of mAb2C4:AF568 (diluted in FACS buffer, 100 *μ*l per well) and resuspended gently. Negative controls are resuspended in FACS buffer only.

The cell suspensions were incubated at 37 °C for 15 min and then annexin V:biotin (1/100 dilution, Trevigen Inc.) was added, followed by gentle mixing and incubation for a further 15 min at 37 °C. The plate was centrifuged, supernatant was removed and streptavidin:AF488 (Thermo Fisher Scientific) was added. The cells were washed again and finally resuspended in 400 *μ*l FACS buffer.
