# Supplementary material for: A novel ligand of calcitonin receptor reveals a potential new sensor that modulates programmed cell death
Source: Cell Death Discov. 2016 Oct 10;2:16062–. doi: 10.1038/cddiscovery.2016.62 (PMC5056446; doi:10.1038/cddiscovery.2016.62)

Supplementary information

Materials and methods

Plasma membrane preparations for the analysis of Cos-7 stable transfectants

Stable transfectants of the monkey kidney cell line Cos-7 were cultured in high glucose Dulbecco's modified Eagle's medium (DMEM; Invitrogen, Carlsbad, CA, USA) plus 10% fetal bovine serum (FBS; Invitrogen) and incubated in a humidified 37°C incubator with 5% CO_2. Polyclonal Cos-7 cell lines, stably expressing cMyc tagged hCTR or a vector control, were derived from the parental line by a combination of selection using 10µg/ml puromycin (Invivogen, Carlsbad, CA, USA) and fluorescence activated cell sorting (FACS) of CTR-positive cells (negative cells in the case of vector control) and then maintained in high glucose DMEM plus 10% FBS with 10 µg/ml puromycin (Invivogen) in a humidified 37°C incubator with 5% CO_2. Transgene expression in all cases was confirmed by flow cytometry (not shown) using the anti-cMyc antibody mAb9E10.

Enriched plasma membranes from parental and hCTR expressing flpIN Cos-7 cells were prepared according to the published protocol [25]. Media was aspirated from cells and cells rinsed with PBS. Cells were harvested in a final volume of 10mL, ice-cold homogenisation buffer (6.6mM imidazole/83mM sucrose pH 7.0 containing 100µM phenylmethane sulfonyl fluoride (Sigma, USA) and 1:1000 dilution of protease inhibitor cocktail (P8340, Sigma, USA)). Cells were disrupted with 3x 10sec bursts of a polytron homogeniser on ice (10mm blade, power setting 3 (Pro Scientific, USA)). Cell homogenate was overlayed on a discontinuous sucrose density gradient consisting of 8mL 60%, 8mL 40%, 8mL 10% sucrose in homogenisation buffer in Sorvall 36mL polyallomer tubes (Thermo Scientific, USA). The sucrose gradient was centrifuged at 23,500rpm (RCF at r_{max} of 102,400) for 3hrs at 4ºC using a Sorvall Surespin 630/36 rotor (Thermo Scientific, USA). Enriched plasma membrane (5mL) was recovered from the 40%/10% interface and diluted with homogenisation buffer to 17mL in Sorvall 17mL polyallomer tubes and pelleted by centrifugation at 30,00rpm (RCF at r_{max} of 166,880) for 30mins at 4ºC in a Sorvall Surespin 630/17 (Thermo Scientific, USA). The final pellet was resuspended in homogenisation buffer and protein concentration assayed by BCA (Pierce, USA).

Protein (60 µg) from a plasma membrane preparation was loaded onto an 8% SDS-PAGE mini-gel and electrophoresed at 100V. Gels were transferred to polyvinylidene difluoride membrane (BioRad, USA) using a semi-dry transfer protocol. After transfer blots were rinsed in PBS-T and blocked with 5%BSA/PBS-T for 30mins. Blots were then incubated for 90mins with primary antibodies as indicated (Figure S1), washed 3x PBS-T,

incubated for 45mins with secondary antibody in dark, washed 3x PBS-T in dark and imaged. Precision molecular weight standards (BioRad, USA) were used to estimate sizes. Primary antibodies (mAb2C4, mAb1H10 and mAb9E10) were used at a concentration of 1µg/mL in 1%BSA/PBS-T plus 0.02% azide. Secondary, goat anti-mouse AF-647 (Molecular Probes, USA) was used at 0.5µg/mL in PBS-T plus 0.02% azide. Immunoblots were imaged on a Typhoon (GE Lifesciences, USA), using 633nM laser and 670/30nM emission filter.

Preparations of whole cell lysates for immunoblot (probed with anti-α-tubulin antibody) analysis of Cos-7 cMyc-human CTR stable transfectants

Cells were grown to 80% confluence. Cells were detached with 5mL Versene per flask, 5mL 1%BSA/PBS added and the cells centrifuged at 300g for 10 minutes. The pellet was washed again in PBS. An aliquot of 1mL of lysis buffer/flask is used to resuspend the pellet. The lysis buffer (10mL) contained 50mM Tris (pH8), 150mM sodium chloride, 2mM EDTA, 1mM PMSF, 1:100 SIGMA II phosphatise inhibitor cocktail, 1mM sodium fluoride and 1 tablet of protease inhibitor cocktail (Complete Mini, Roche). The cells were disrupted using an Ultra Turrax (IKA, Germany) homogeniser on the power setting of 4.5 (6=maximum) for 30 seconds at RT. The homogenate was centrifuged (low speed spin) in 1.5mL tubes at 5000RPM for 10 minutes in a microfuge. The supernatant was centrifuged at 100,000g in an XL-90 Beckman ultracentrifuge for 60 minutes set at 4°C and the pellet resupended in 10µL/flask of lysis buffer (described above). Aliquots were stored at -80°C. Protein was determined BCA protein assay kit (ThermoScientific, US).

Samples were solubilized in preparation buffer (60mM Tris [pH6.8], 2% SDS, 10% glycerol, 2.5% β-mercaptoethanol, 0.01% bromophenol blue), heated for 5 minutes at 50°C, placed on ice prior to loading onto the PAGE-SDS acrylamide gel (3% stacking/8% resolving) and the apparent molecular weight estimated using Page Ruler (BioRad, San Diego) pre-stained protein standards . The gel was run initially at 100volts until the dye moved into resolving gel, then increased to 180volts and run for 40 minutes. The resolved proteins were transferred from the 1.5mm gel (semi-dry blot, BioRad Transblot SD) onto 0.2µm PVDF membrane (BioRad, US) over 1 hour at 15volts.

The identification of protein bands was achieved using the Pierce ECL protocol (ThermoScientific, Rockford,US) and the final concentration of primary mouse anti-α-tubulin antibody (0.23 µg/mL, Sigma T6074) ,and secondary antibody, goat polyclonal anti-mouse/HRP (1:7000 of DAKO P0447). The final ECL substrate solution

(Pierce/ThermoScientific, Rockford,US) was incubated with the membrane for 1-2 minutes and the bands detected using a LAS 3000 Chemiluminescence detector (FujiFilm).

Preparation of cytosolic proteins, nuclear proteins and membrane proteins for immunoblots

The cells were grown in a 75cm2 tissue culture flask until approx. 80% confluency. The cells are rinsed gently with 3ml versene, followed by incubation at 37C for 5 mins with 5 ml versene. Once detached the cells were transferred to a 50ml tube and centrifuged for 5 mins at 1300rpm. The cell pellets were resuspended in 1ml PBS and then transferred to 1.5ml microfuge tubes on ice, followed by centrifugation at 4C for 5 mins at low speed (350 x g). The pellets were resuspended in 2-3 volumes of cold hypotonic buffer (10mM HEPES, pH 7.4, 1.5mM MgCl2, 10mM NaCl, 10% glycerol) with fresh protease inhibitors, 1mM DTT and 1mM PMSF (about 250ul). The samples were incubated for 20-30 mins at 4C to swell the cells. At this stage the samples can be put at -80C for at least 60 minutes or as long as weeks, or snap frozen in ethanol/dry ice or liquid nitrogen. The samples are then thawed on ice and centrifuged at maximum (16,300 x g) for 30 minutes at 4C. The supernatant is the cytosolic fraction which was removed and stored at -80C. The pellets were resuspended in 3/5 volume of cold hypertonic buffer (10mM HEPES, pH7.4, 1.5mM Mgcl2, 420mM NaCl, 10% glycerol) with fresh protease inhibitors, 1mM DTT and 1mM PMSF (about 60ul). These were incubated at 4C for 30 minutes and then centrifuged at maximum (16,300 x g) at 4C for 30 mins. The supernatant is the nuclear protein fraction which was removed and stored at-80C. The remaining pellets were resuspended in 3/5 volume of RIPA buffer (10mM phosphate buffer, pH 7.2, 150mM NaCl, 1% NP-40, 1% Sodium deoxycholate, 0.1% SDS, 0.2mM EDTA, 50mM NaF) with fresh protease inhibitors, 1mM DTT and 1mM PMSF (about 60ul). These were incubated at 4C for 10 mins followed by centrifugation at 16,300 x g at 4C for 20 mins. The supernatant is the membrane extract, which was transferred to a fresh microfuge tube on ice. This extract is best used fresh but may be stored at -80C.

Methods for qPCR

Hct116 and hct116bak$^{-/-}$bax$^{-/-}$ cell lines were cultured in media alone or supplemented with 50µM etoposide or 0.5µM staurosporine for 48 hours. The percentage of cell death for the cell lines hct116 and hct116bak$^{-/-}$bax$^{-/-}$ after treatment with staurosporine (50%, 0% respectively) or etoposide (20%, 5% respectively) was calculated by trypan blue inclusion. Cells were harvested and mRNA prepared using RNeasy minikit for cells

(Promega). The quality of the RNA was checked with a spectrometer (OD260/OD280 ratio). cDNA was prepared with SuperScript® VILO™ cDNA Synthesis Kit (Thermo Fisher Scientific). Primers and probes for CTR cDNA (SKU 4331182 –Specify Assay ID - Hs01016882_m1 FAM MGB) and 18S cDNA control (SKU 4448484 –Specify Assay ID - Hs03928990_g1 VIC MGB Primer Limiting) were purchased from Applied Biosystems. Assays were performed in 96-well plates using Taqman gene expression kits (Applied Biosystems). Each 96-well plate qPCR assay was performed in triplicate (staurosporine) or quadruplicate (etoposide) on separate occasions on an ABI 7500 real time PCR machine. The final values are calculated from $2^{-\Delta\Delta CT}$ x 100 and normalised for each cell line against untreated controls.

Legends

Figure S1: Characterization of antibodies conjugated with fluorophores and carboxyl terminal sequences of GPCRs indicating arginines and PDZ domains that interact with tubulin.

A. Gel electrophoresis/immunoblots of enriched membrane preparations from Cos-7 vector control (lanes 2, 4, 6) and Cos-7 cMyc-human CTR (lanes 3, 5, 7) cell lines and total cell lysates of Cos-7 vector control (lane 9) and Cos-7 cMyc-human CTR (lane 8) cell lines. The immunoblots were developed using anti-CTR antibodies mAb2C4 (lanes 2 & 3), mAb1H10 (lanes 4 & 5), an anti-cMyc antibody mAb9E10 (lanes 6 & 7) and anti-α tubulin antibody (lanes 8 & 9). MAb1H10 have been validated with additional data elsewhere [25].

B. Gel electrophoresis/immunoblots of cytosolic proteins (lanes 2 & 5, 70µg/lane), nuclear proteins (lane 3, 50µg/lane) and membrane proteins (lanes 4 & 6, 70µg/lane) from the cell lines COS-7 (lanes 2 – 4) and MG63 (lanes 5 & 6), probed with the antibody mAb2C4. A band of approximately 55kD is clearly concentrated in the cytosolic fraction which is likely to include small endosomes.

C. FACS analysis of Cos-7 (cMyc-tagged) human CTR (■) and Cos-7 vector control (■) with the anti-hCTR antibody MAb2C4 and, D. MAb9E10. The black histogram in C & D represents unstained cells.

E. The reactivity of the conjugate (mAb9E10:AF647**)** with the cMyc sequence was assayed using live cell staining with 2µg/mL conjugated antibody and flow cytometry of Cos7 cells stably transfected with vector alone or cMyc tagged human CTR. Confirmation that the negative control mAb9E10:AF647 conjugate still recognises the cMyc epitope is shown here, in which vector control cells are represented with the black histogram and stained cMyc tagged CTR expressing cells in grey. mAb9E10:AF647 is the isotype control in Fig S4.

E**.** The alignment of type 2 PDZ sequences within the carboxyl domains of CTR from several species.

F. The alignment of arginine sequences within the carboxyl domain of the α_{2B} adrenergic receptors that bind α-tubulin (R_{437}, R_{441}, R_{446}) [49] with similar sequences within CTR from several mammalian species.

Figure S1

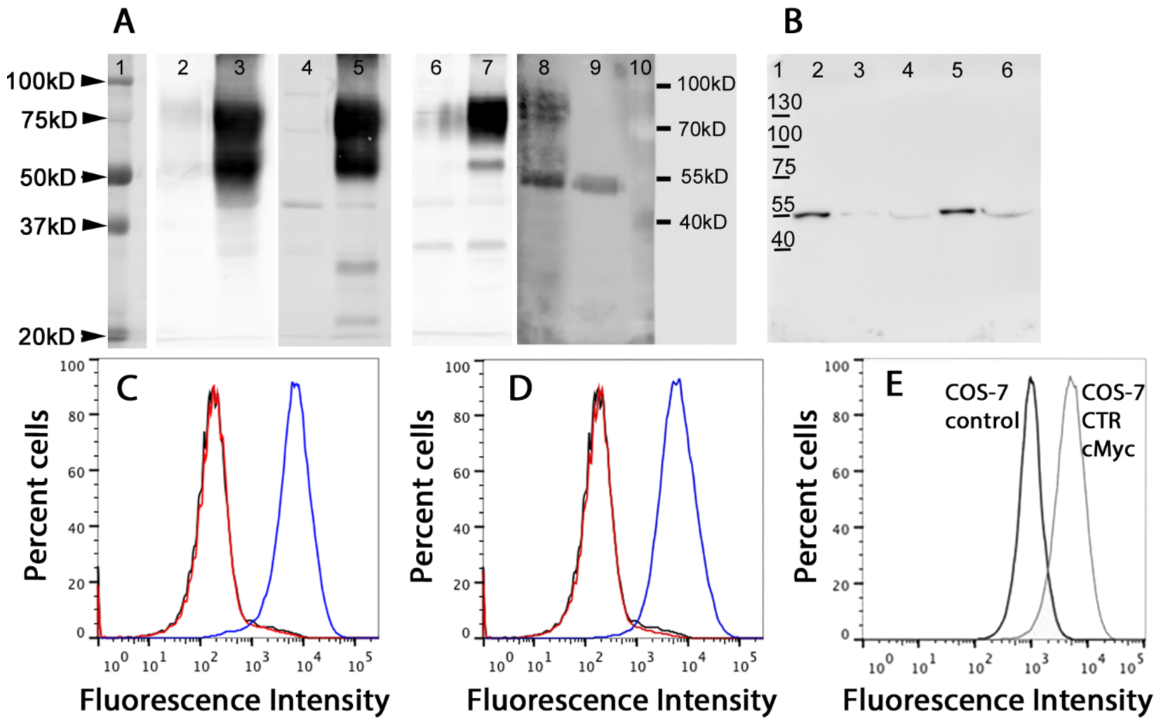

F Type 2 PDZ docking site conserved in all CTR sequences identified so far (consensus is D/E-S/T-X-Φ where Φ is hydrophobic and usually Alanine, Valine, Leucine or Isoleucine

Homo sapiens	-NQGEESAEII	PLNIIEQ---	ESSA	474
Mus musculus	-NEGEESTEMI	PMNVIQQ---	DASA	478
Rattus norvegicus	-NEGEESTEMI	PMNVIQQ---	DSSA	496
Gallus gallus	-RYVDDSELVA	LKSG----------	ETSA	469
Sus scrofa	-PVVEVEGVEV	IAMEVLEQ--	ETSA	482
Jaculus jaculus	-NQVLEGAEII	PLNTVEQ---	ESSA	476
Xenopus tropicalis	-KYGDESEITA	LNSG------	DTYA	478

G Arginine sequences in the carboxyl termini of receptors that interact with a tubulin

Homo sapiens α2B adrenergic receptor	NSSLNPVIYT	IFNQDF**RR**AF	**RR**ILC**R**PWTQ	TAW
Rattus rattus α2B adrenergic receptor	NSSLNPVIYT	VFNQDF**RR**AF<br>**R437**	**RR**ILC**R**PWTQ<br>**R441 R446**	TGW
Homo sapiens calcitonin receptor	QTTVKRQWAQ	FKIQWNQ**R**GW	**RR**PSN**R**SA**R**A	A

Figure S2: Live staining and uptake of mAb2C4:AF568 by apoptotic MG63 cells and images of U87MG cells treated with 1% DMSO and 1µM paclitaxel

In (A) MG63 cells treated with staurosporine for 19 hours. Live staining with 1µg/mL mAb2C4:AF568 for 0 minutes and 60 minutes is shown. At zero minutes fluorescence is associated with the cell membrane whereas at 60 minutes fluorescence has become concentrated in the perinuclear region. The method used for live staining is described in the methods section. In (B-F) U87 MG cells are treated as shown for MG63 cells (Figure 2 M-Q) with 1% DMSO plus 1µM paclitaxel for 19 hours. U87 MG cells that express α-tubulin are typically small with low residual uptake of mAb2C4:AF568 (arrowheads in C & D). On the other hand larger cells (examples with arrows) that have inclusions bright with mAb2C4:AF568 are negative for α-tubulin and are apoptotic. The calibration bar shown in (F) represents (A) 2.5µm and (B-F) 80µm.

Figure S2

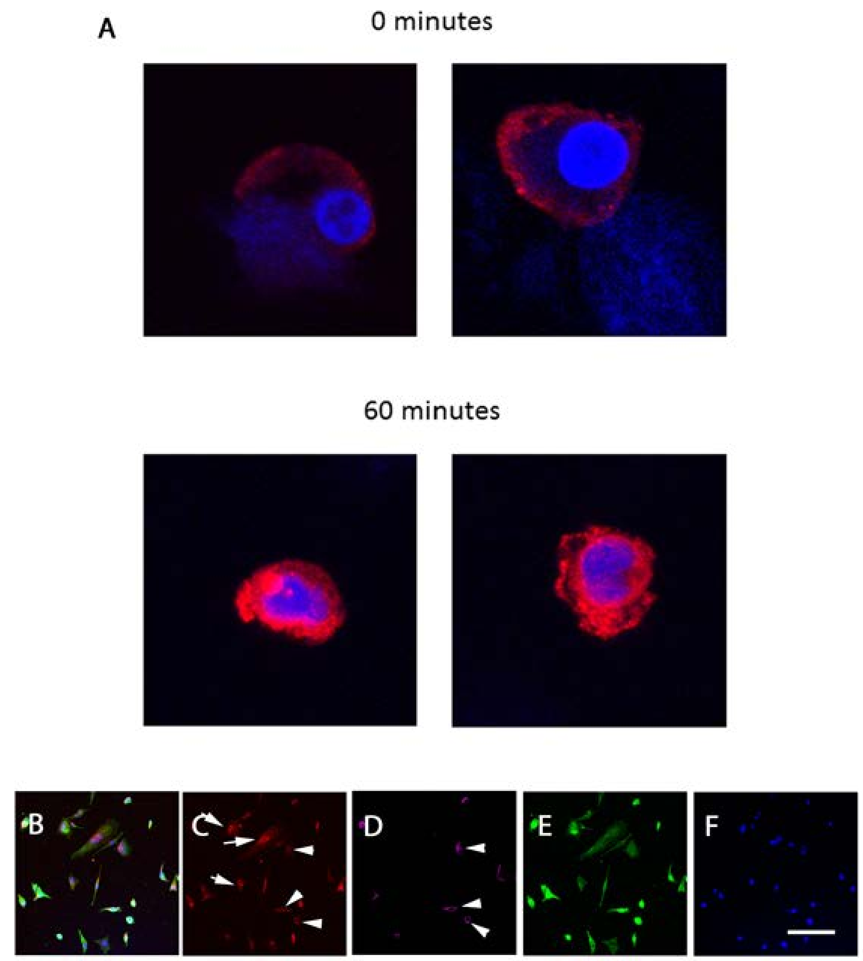

Figure S3: Data from flow cytometry of cytotoxin-treated Jurkat cells, untreated Cos-7 stable transfectants and untreated HEK293 stable transfectants

A. Characterisation of the mAb2C4:AF568 compared to an isotype control mAbFLAG:AF568 in Jurkat cells treated with 5 X 10^{-7}M staurosporine. Trace 1, mAbFLAG:AF568 alone; trace 2, mAb2C4:AF568 in the presence of annexin V:AF488; trace 3, mAb2C4:AF568 alone; trace 4, annexin V:AF488 alone; trace 5, not stained with fluorophores.

B. Characterisation of the mAb2C4:AF568 in a Cos-7 stable transfectant expressing CTR (traces 1 & 3) compared or the Cos-7 stable transfectant vector control (traces 2 & 4).Traces 1& 2 represent staining with 1µg/mL mAb2C4:AF568 and traces 3 & 4 were unstained.

C. Characterisation of the mAbFLAG:568 antibody (isotype control) compared to mAb2C4:AF568 in HEK293 stable transfectant that does not express CTR but a PA Receptor:FLAG surface protein. Trace 1 (black) represents no staining; trace 2 (red), stained with 1µg/mL MAb2C4:AF568 and trace 3 (blue) stained with 1µg/mL mAbFLAG:AF568.

Figure S3

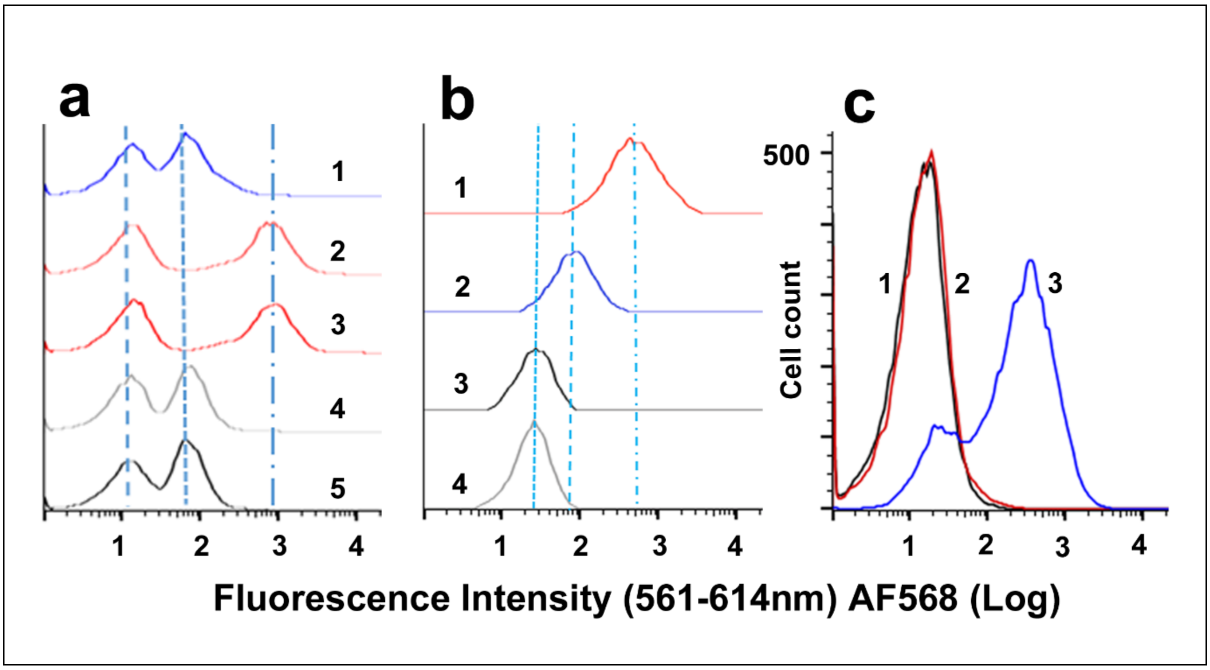

Figure S4: Confocal images of other markers and controls for MG 63 cells.

MG 63 cells treated with 1µM staurosporine for 19 hours in several separate experiments. Staining with mAb2C4:AF568 post-fixation (A**)** [X63] and **(**B**)** [X20]. (C) Merged image of live staining with mAb9E10:AF647 (IgG1 isotype control, pink-negative colour) and annexin V (green). (D-G) Live staining with mAb2C4:AF568: (D) DAPI, (E) mAb2C4:AF568, (F) annexin V and (G) post-fix staining with anti-caspase 3 antibody. (H-I) Live staining with mAb2C4:AF568: (H) merged, stained post-fixation with anti-LAMP-1 (green) antibody, (I) mAb2C4:AF568. (J-M) Live staining with mAb2C4:AF568: (J) DAPI; (K) mAb2C4:AF568; (L) annexin V:AF488; and (M) post-fix staining with anti-caspase 8 antibody. (N-Q) Live staining with mAb2C4:AF568: (N) DAPI, (O) mAb2C4:AF568, (P) annexin V and (Q) post-fix staining with anti-caspase 9 antibody. **(**R-U**)** show images (X20 magnification) of cells treated with 1% DMSO alone: (R) merged; (S) mAb2C4:AF568; (T) α-tubulin; (U) DAPI. All panels represent live staining with 10µg/mL mAb2C4:AF568 except (C) in which 10µg/mL mAb9E10:AF647) was used and (A, B) in which staining was performed with mAb2C4:AF568 post-fixation. Calibration bar shown in (U) represents in (A, D-I), 30µm; (B, C), 50µm; (J-Q), 20µm; (R-U), 60µm.

Figure S4

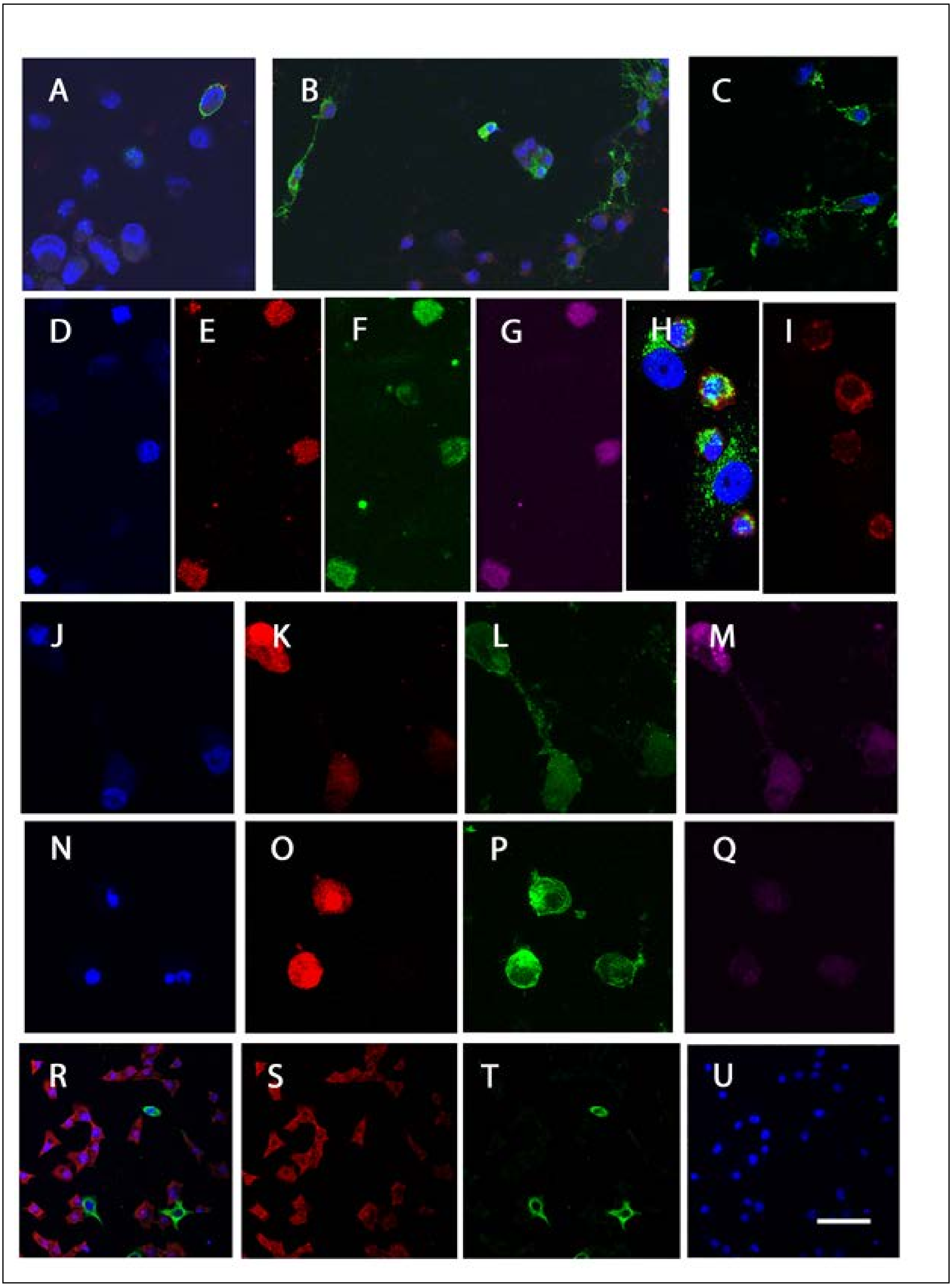

Figure S5: The induction of CTR mRNA measured by qPCR. The cell line hct116 and a mutant hct116 bak$^{-/-}$bax$^{-/-}$ were treated with two different cytotoxins, staurosporine (0.5µM) or etoposide (50µM). These cytotoxins induce apoptosis in the hct116 cell line but not in the mutant. The relative, normalised values for CTR mRNA ± standard errors are: hct116 (A: untreated), 99.9±5.3; B:staurosporine, 129.7±5.3; C: etoposide, 134.6±13.8; hct116 mutant (D: untreated), 99.8±2.8; E: staurosporine, 137.8±4.5; F: etoposide, 177.3±17.9.

Figure S5

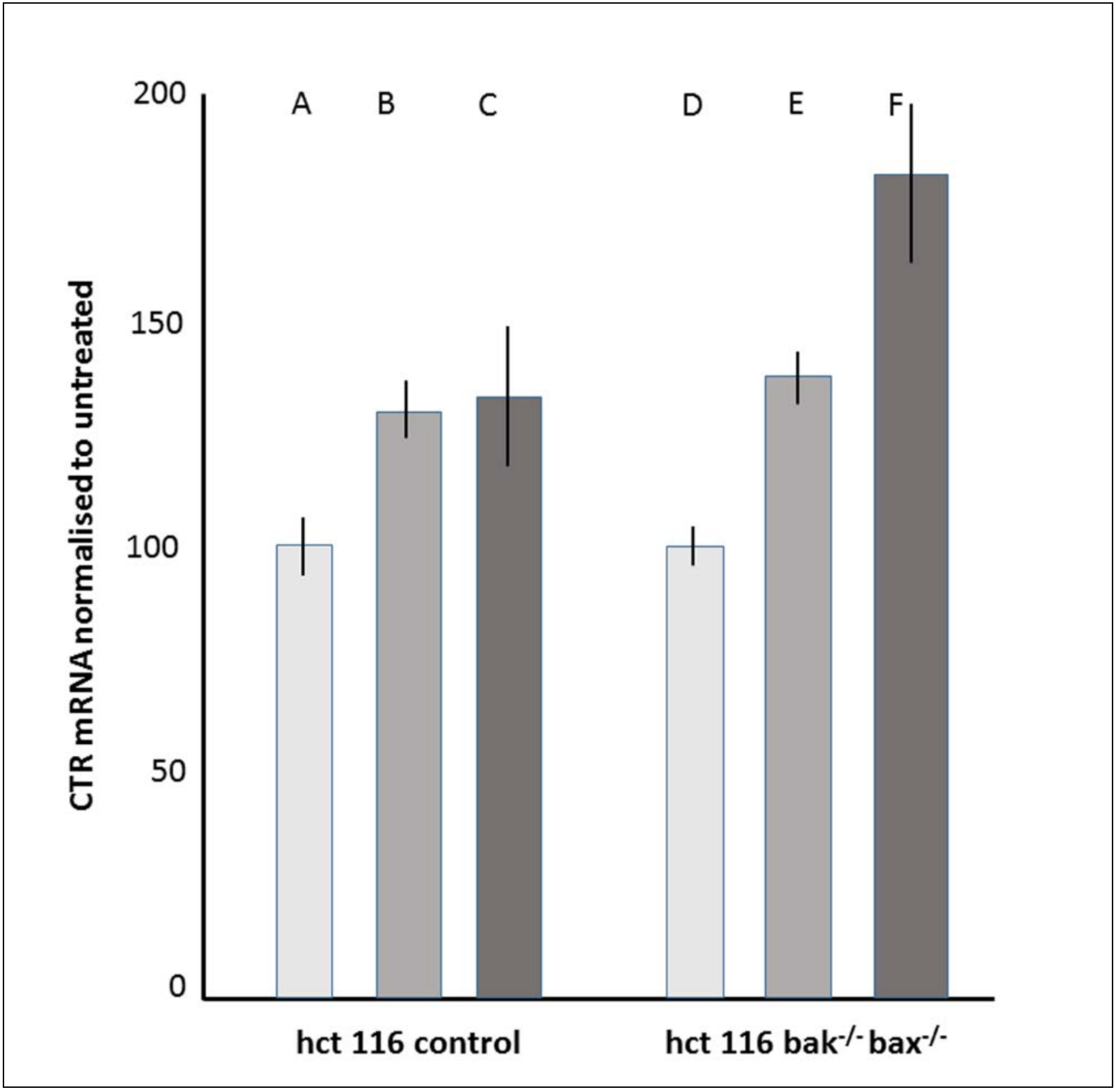

Supplement: Supplementary Information [file cddiscovery201662-s1.pdf]
